# Trastuzumab-induced thrombocytopenia after eight cycles of trastuzumab treatment

**DOI:** 10.1515/med-2020-0201

**Published:** 2020-07-13

**Authors:** Qiong Zhou, Jie Dong, Xiaodong Jiang, Yueyin Pan, Xinghua Han

**Affiliations:** Department of Oncology, Provincial Hospital affiliated to Anhui Medical University, Hefei, Anhui Province, 230032, China; The First Affiliated Hospital of USTC, Division of Life Sciences and Medicine, University of Science and Technology of China, Hefei, Anhui Province, 230001, China

**Keywords:** trastuzumab, thrombocytopenia, breast cancer

## Abstract

Trastuzumab, a humanized monoclonal antibody derived from recombinant DNA, is used in patients with breast cancer with HER2 gene amplification. The survival benefit from trastuzumab has been well established in patients with early and metastatic breast cancer who had over expression of HER2. We reported a case of severe thrombocytopenia after eight cycles of trastuzumab treatment for breast cancer. Before the 9th trastuzumab treatment, the patient’s platelet decreased to 48 × 10^9^/L. Recombinant human thrombopoietin was used, and the platelet level increased to normal level. Before the 10th treatment, the platelet count of the patient was 99 × 10^9^/L. However, during the 10th and 11th trastuzumab treatment, the platelet count decreased to 5 × 10^9^/L in 24 h. After treatment with TPO and corticosteroids, the platelet levels increased to the normal level in 7 days. Trastuzumab-induced thrombocytopenia is rare but still occurred even after 8 cycles of trastuzumab treatment.

## 1 Introduction

Drug-induced thrombocytopenia (DIT) can be evoked by a variety of medications. Although their clinical manifestations vary, severe thrombocytopenia can develop fatal bleeding symptoms [1]. In cancer patients, it can be easily ignored by physicians because of chemotherapy-induced myelosuppression or tumor bone marrow infiltration, especially in patients with bone metastasis or adjuvant chemotherapy. DIT-related drugs have been reported to contain chemotherapeutic agents such as oxaliplatin [2], irinotecan, fludarabine [3], and monoclonal antibodies such as abciximab, infliximab, and rituximab [1], but trastuzumab-induced thrombocytopenia is very rare. This study reported a patient with life-threatening thrombocytopenia after long-term trastuzumab infusion.

## 2 Case report

A 35-year-old woman presented with a 3-month history of waist and back pain. Examination revealed a tumescent left supraclavicular lymph node (no mass was touched in the breast). Imaging revealed multiple hypermetabolic foci of FDG in the proximal axial bone and appendage bone as well as multiple and tumescent lymph nodes on the bilateral pulmonary hilum. There was no sign of viscera metastases. Core biopsy showed poorly differentiated metastatic breast carcinoma that was estrogen receptor positive, progesterone receptor negative, and strongly human epidermal growth factor receptor (HER2) positive (3+).

Palliative chemotherapy with docetaxel (75 mg/m^2^), cisplatin (75 mg/m^2^), and trastuzumab (6 mg/kg, first treatment using 8 mg/kg) was started for 6 cycles, followed by two cycles of docetaxel and trastuzumab treatment. After the treatment, CT scanning showed that all the lymph nodes disappeared and symptoms of pain all over the body had also been relieved. Nevertheless, the patient’s platelet count decreased to 48 × 10^9^/L before the 9th treatment. Docetaxel-induced bone marrow suppression was suspected, and trastuzumab and capecitabine (1.5 g, bid, 1–14 d) were used for the 9th treatment. Meanwhile, recombinant human thrombopoietin (TPO) was injected to increase the platelet level. As anticipated, the platelet count returned to normal in 3 days.

**Figure 1:**
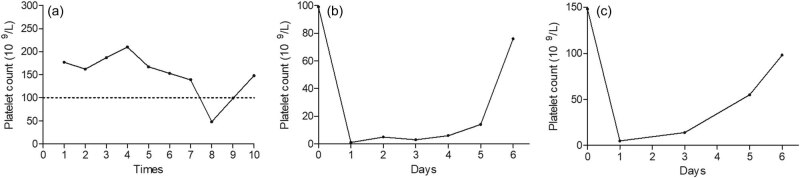
Platelet count changes during trastuzumab therapy: (a) before each trastuzumab infusion; (b) during the 10th infusion of trastuzumab; and (c) during the 11th infusion of trastuzumab.

Before the 10th treatment, the platelet count of the patient was 99 × 10^9^/L (Figure 1a). Trastuzumab and capecitabine were used again for the 10th treatment. The patient felt tiredness and nausea and all-over body petechiae, and gums bleeding occurred after a 6 h trastuzumab infusion. Blood examination showed that the platelet count was 1 × 10^9^/L. Blood smear examination showed severe thrombocytopenia with no schistocytes, spherocytes, clumps, and giant platelets. Bone marrow biopsy showed normal megakaryocytopoiesis without signs of tumor infiltration. Heparin had never been used and other causes of thrombocytopenia such as disseminated intravascular coagulation, thrombotic thrombocytopenic purpura, hereditary thrombocytopenia, and hemolysis were excluded because of her normal clotting, D-dimers, immunoglobulins, hemoglobin, renal function, bilirubin, and lactate dehydrogenase as well as lack of schistocytes. After consultation with the hematologist, drug-induced thrombocytopenia was suspected. Given the treatment effect of trastuzumab, capecitabine was withdrawn and 3 weekly trastuzumab was maintained. After treatment with platelet transfusion, TPO, and corticosteroids, the platelet count returned to the normal level (Figure 1b). Trastuzumab alone was used for the 11th treatment. Dental bleeding, diffuse petechiae and ecchymosis occurred again in 24 h after the infusion and the platelet count decreased to 5 × 10^9^/L (Figure 1c). The new bone marrow biopsy results were consistent with the previous one. After reviewing the treatment process and referring to relevant literature, a diagnosis of trastuzumab-induced thrombocytopenia was finally made. After treatment with platelet transfusion, TPO and corticosteroids, the platelet count returned to normal.

**Consent:** Written consent was obtained from the patient for publication of this case report.

## 3 Discussion

According to the criteria proposed by George et al. to establish the relationship between a drug and thrombocytopenia [4], this patient was diagnosed with trastuzumab-induced thrombocytopenia. Cathomas et al. first reported trastuzumab-induced thrombocytopenia in a letter to the editor of *New England Journal of Medicine* in 2007 [5], while the mechanisms of trastuzumab-induced thrombocytopenia was still unclear. Drug-induced thrombocytopenia can occur as a result of direct bone marrow toxicity via impaired megakaryocyte proliferation and maturation or via immune-mediated peripheral destruction of platelets [14]. Our and previous cases all had shown a normal megakaryocytopoiesis by bone marrow biopsy [5,6,7,8,9,10,11,12,13]. In addition, Uppal et al. showed that trastuzumab neither directly affected platelet activation and aggregation, nor inhibited megakaryocyte differentiation [15]. Thus, trastuzumab-induced immune-mediated peripheral destruction of platelets may be the reason to thrombocytopenia.

As Table 1 showed, thrombocytopenia can occur after first exposure to trastuzumab or several cycle of trastuzumab treatment. In our article, thrombocytopenia occurred after eight cycles of trastuzumab treatment. To our knowledge, it was the longest treatment of trastuzumab before thrombocytopenia. Previous studies have suggested that thrombocytopenia caused by a first exposure to trastuzumab is related to naturally occurring antibodies that recognize the murine structural elements of trastuzumab. These antibodies bind to platelets and result in platelet clearance [8,12]. In the case of thrombocytopenia with a delayed exposure to trastuzumab, our hypothesis was that antibodies caused by first exposure to trastuzumab were insufficient. Thrombocytopenia may not occur until sufficient antibodies were produced. This speculation can also explain the relatively higher platelet nadir after the 8th treatment.

**Table 1:** Review of patients with severe thrombocytopenia induced by trastuzumab

Cases	Age (years)	sex	HRs status	Exposure time(s)^a^	Clinical manifestation	Concurrent chemotherapy	Platelet Nadir ( × 10^9^/L)	Management
Cathomas [5], 2007	54	F	−	1	Petechiae	No	3	IVIGs
					Nose bleeding			Corticosteroids
Parikh et al. [6], 2008	56	F	+	1	Tiredness nausea	No	2	IVIGs
					Petechial rash			
Drudi et al. [7], 2009	NM	F	NM	1	Ecchymosis	Docetaxel	7	PLT IVIGs
								Corticosteroids Dexamethasone
Jara Sanchez et al. [8], 2009	37	F	+	1	Petechiae	Docetaxel	3	PLT IVIGs
						Carboplatin		Corticosteroids
								Dexamethasone
								Splenectomy
Mantzourani et al. [9], 2011	56	F	−	1	Petechiae	No	5	IVIGs
					Nose bleeding			
Pino et al. [10], 2013	70	F	+	2	Ecchymosis	Vinorelbine	2	PLT IVIGs
								Corticosteroids
Aguirre et al. [11], 2013	63	F	+	NM	Petechiae	Paclitaxel	22	Corticosteroids
Zeng et al. [12], 2014	57	F	+	2	Tiredness	Abraxane	28	Etamsylate
						Carboplatin		TPO
Miarons et al. [13], 2016	70	F	−	4	None	Docetaxel	39	Corticosteroids
Present case	35	F	+	8	Petechiae	Capecitabine	1	TPO PLT
					Nose bleeding			Corticosteroids

^a^First instance of thrombocytopenia occurred after exposure to trastuzumab; F, Female; HR, Hormone receptor; IVIGs, intravenous immunoglobulin; TPO, thrombopoietin; NM, not mentioned; PLT, platelet transfusion.

Trastuzumab is an efficacy agent in breast cancer with HER2 gene amplification and can reduce 50% of risk of recurrence [16]. All but two cases discontinued trastuzumab after thrombocytopenia. One of these two cases continued because thrombocytopenia did not reappear after two cycles of trastuzumab treatments [9]. The other case continued by prolonging the interval between trastuzumab treatments, alleviating thrombocytopenia [12]. Steroids can prevent platelet removal by the reticuloendothelial system, reduce the levels of platelet-associated antibodies and increase platelet production by the bone marrow [17]. IVIG can block the Fc-receptor on macrophages of the reticuloendothelial system. This prevents phagocytosis of antibody-coated platelets and therefore inhibits platelet destruction [18]. TPO can increase platelet production by the bone marrow. Thus, these medications were used to treat drug-induced thrombocytopenia. In our case, a marked acceleration of thrombocytopenia occurred after the 10th treatment. However, the thrombocytopenia did not occur after the 9th trastuzumab treatment. The reason may be preventively using TPO. Whether preventively using TPO or other drugs such as corticosteroids and IVIG could prevent or alleviate trastuzumab-induced thrombocytopenia is worth studying.

## Abbreviations

DIT: drug-induced thrombocytopenia
HER2: human epidermal growth factor receptor
TPO: recombinant human thrombopoietin
IVIG: intravenous immunoglobulin

## References

[1] Aster RH, Curtis BR, McFarland JG, Bougie DW. Drug-induced immune thrombocytopenia: pathogenesis, diagnosis, and management. J Thrombosis Haemostasis: JTH. 2009;7:911–8. 10.1111/j.1538-7836.2009.03360.x PMC2935185 19344362

[2] Erdem GU, Dogan M, Demirci NS, Zengin N. Oxaliplatin-induced acute thrombocytopenia. J Cancer Res Therapeutics. 2016;12:509–14. 10.4103/0973-1482.154056 27461601

[3] Jiang Y, Peng H, Cui X, Zhou Y, Yuan D, Sui X, et al. Autoimmune thrombocytopenia: a complication of fludarabine therapy in the treatment of Waldenstrom’s macroglobulinemia. Int J Clin Exp Med. 2014;7:5937–42. PMC4307585 25664138

[4] George JN, Raskob GE, Shah SR, Rizvi MA, Hamilton SA, Osborne S, et al. Drug-induced thrombocytopenia: a systematic review of published case reports. Ann Intern Med. 1998;129:886–90. 10.7326/0003-4819-129-11_part_1-199812010-00009 9867731

[5] Cathomas R, Goldhirsch A, von Moos R. Drug-induced immune thrombocytopenia. N Engl J Med. 2007;357(18):1870–1. 17985437

[6] Parikh O, Neave F, Palmieri C. Severe thrombocytopenia induced by a single infusion of trastuzumab. Clin Breast Cancer. 2008;8:285–6. 10.3816/CBC.2008.n.034 18650161

[7] Drudi F, Gianni L, Fantini M, Ravaioli A. Trastuzumab-related thrombocytopenia: always a self-limiting complication? Ann Oncol. 2009;21:668–9. 10.1093/annonc/mdp566 20032121

[8] Jara Sanchez C, Olier Garate C, Garcia-Donas Jimenez J, Penalver Parraga J. Drug-induced thrombocytopenia induced by trastuzumab: a special challenge in a curable disease. Ann Oncol. 2009;20:1607–8. 10.1093/annonc/mdp374 19633054

[9] Mantzourani M, Gogas H, Katsandris A, Meletis J. Severe thrombocytopenia related to trastuzumab infusion. Med Sci Monitor: Int Med J Exp Clin Res. 2011;17:Cs85–87. 10.12659/MSM.881838 PMC3539572 21709639

[10] Pino MS, Angiolini C, Fioretto L. Severe thrombocytopenia after trastuzumab retreatment: a case report. BMC Res Notes. 2013;6:400. 10.1186/1756-0500-6-400 PMC3851485 24093447

[11] Aguirre E, Taberner T, Luana A, Morales S, Llombart A. Severe thrombocytopenia related to long-term trastuzumab exposure. Tumori. 2013;99:e1–2. 10.1177/030089161309900122 23549016

[12] Zeng R, Dai X, Xie F, Chen E, Qu J, Hu X. Severe thrombocytopenia induced by second rxposure to trastuzumab can be alleviated by prolonging the interval between treatments. Clin Breast Cancer. 2014;14:e69–72. 10.1016/j.clbc.2013.11.004 24388532

[13] Miarons M, Velasco M, Campins L, Fernández S, Gurrera T, Lopez-Viaplana L. Gradual thrombocytopenia induced by long-term trastuzumab exposure. J Clin Pharm Therapeutics. 2016;41:563–5. 10.1111/jcpt.12416 27425556

[14] Aster RH. Drug-induced immune thrombocytopenia: an overview of pathogenesis. Semin Hematol. 1999;36:2–6. 9930556

[15] Uppal H, Doudement E, Mahapatra K, Darbonne WC, Bumbaca D, Shen BQ, et al. Potential mechanisms for thrombocytopenia development with trastuzumab emtansine (T-DM1). Clin Cancer Res. 2015;21(1):123–33. 10.1158/1078-0432.CCR-14-2093 25370470

[16] Baselga J, Carbonell X, Castaneda-Soto NJ, Clemens M, Green M, Harvey V, et al. Phase II study of efficacy, safety, and pharmacokinetics of trastuzumab monotherapy administered on a 3-weekly schedule. J Clin Oncol. 2005;23:2162–71. 10.1200/JCO.2005.01.014 15800309

[17] Gernsheimer T, Stratton J, Ballem PJ, Slichter SJ. Mechanisms of response to treatment in autoimmune thrombocytopenic purpura. N Engl J Med. 1989;320(15):974–80. 10.1056/NEJM198904133201505 2927480

[18] Ray JB, Brereton WF, Nullet FR. Intravenous immune globulin for the treatment of presumed quinidine-induced thrombocytopenia. DICP. 1990;24:693–5. 10.1177/106002809002400706 1695793

